# Evidence based treatment for lupus nephritis: present perspectives and challenges

**DOI:** 10.3389/fneph.2024.1417026

**Published:** 2024-08-06

**Authors:** Efstathios Xagas, Konstantinos Drouzas, George Liapis, Sophia Lionaki

**Affiliations:** ^1^ Department of Nephrology, 2^nd^ Department of Propaedeutic Internal Medicine, Attikon University Hospital, Medical School, National and Kapodistrian University of Athens, Athens, Greece; ^2^ 1^st^ Department of Pathology, Medical School, National and Kapodistrian University of Athens, Athens, Greece

**Keywords:** lupus nephritis, therapy, outcome, histopathology, management

## Abstract

Systemic lupus erythematosus (SLE) is a complex autoimmune disease known for its high heterogeneity among individuals, which affects various organs including the kidneys. Lupus nephritis (LN) is a frequent and life-threatening manifestation of the disease, with up to 50% of patients developing kidney involvement. Classification of renal involvement in lupus is based on specific histopathological findings, guiding therapeutical decisions. Immunosuppressive therapy, particularly glucocorticoids combined with cyclophosphamide or mycophenolate mofetil, has been the mainstay of treatment for many years, while rates of complete remission have not changed dramatically. Despite advancements in therapy, in an important proportion of patients LN leads to end-stage kidney disease (ESKD). Emerging therapies including belimumab, voclosporin, and obinutuzumab offer promising results in improving renal outcomes, especially in refractory or relapsing disease. Maintenance therapy is crucial to prevent disease flares and preserve renal function. Supportive measures including lifestyle modifications and non-immunosuppressive pharmacological interventions are nowadays also essential in managing LN. This review emphasizes recent advances of therapy and challenges regarding treatment optimization with strategies to improve long-term outcomes.

## Introduction

Systemic lupus erythematosus (SLE) is an autoimmune disease known for its complexity and the exhibition of high heterogeneity among affected individuals. Lupus pathogenesis is mainly linked with the production of autoantibodies. The mechanism of autoantibodies creation includes a blend of genetic, epigenetic, hormonal, immunoregulatory and environmental factors. Heterogeneity is reflected to varied clinical manifestations related to the organs affected as well as to the way the disease manifests in a specific organ (1).

Kidney involvement in SLE is a frequent manifestation of the disease and can be evolved to a life-threatening condition. Kidney involvement is observed in about 50% of patients with SLE and from this percentage, up to 10% will develop end-stage kidney disease (ESKD). It is remarkable that LN itself has been linked with an threefold increased risk of death (2, 3). LN is the most common form of kidney involvement, although SLE can cause kidney injury in various other ways including tubulointerstitial disease (4), lupus podocytopathy (5), and vascular involvement i.e. thrombotic ic microangiopathy (6) or vasculitis (7). Lupus nephritis is defined as a glomerular injury being developed in patients with SLE and is associated with the detection of stains for IgG, IgM, C3 and C1q in immunofluorescence. There are different types of renal involvement in lupus, manifesting with various clinical signs and diverse prognoses (8). Certain patient characteristics, including black race, male sex, pediatric onset, frequent relapses, incomplete remission and high proteinuria, have been associated with greater risk for progressive kidney disease and renal failure (9).

Although the prognosis of renal involvement in patients with SLE has significantly improved over the past few decades, a high percentage of patients still progress to ESKD. Therefore, there is a well-defined need for new insights in the management of LN with the introduction of targeted immunosuppressive therapies in addition to supportive and non-specific interventions to attenuate chronic kidney disease (CKD) progression.

## Histopathology

**Figure 1:**
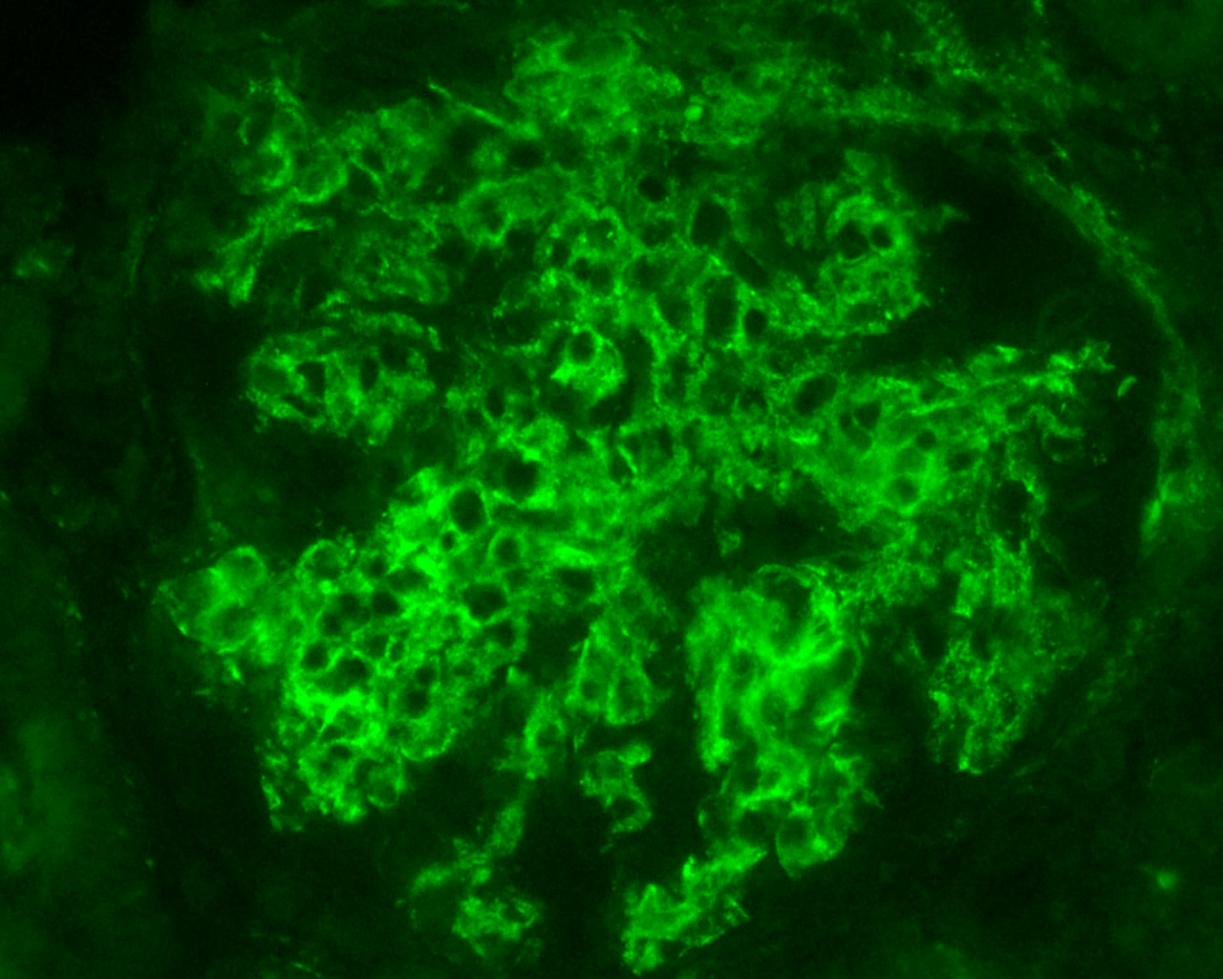
Intense C1q staining, in mesangium and segmentally in glomerular basement membranes, from a case of lupus nephritis, Class IV (C1q X 400, Immunofluorescence examination, DAKO FITC, 1/50 dilution).

**Figure 2:**
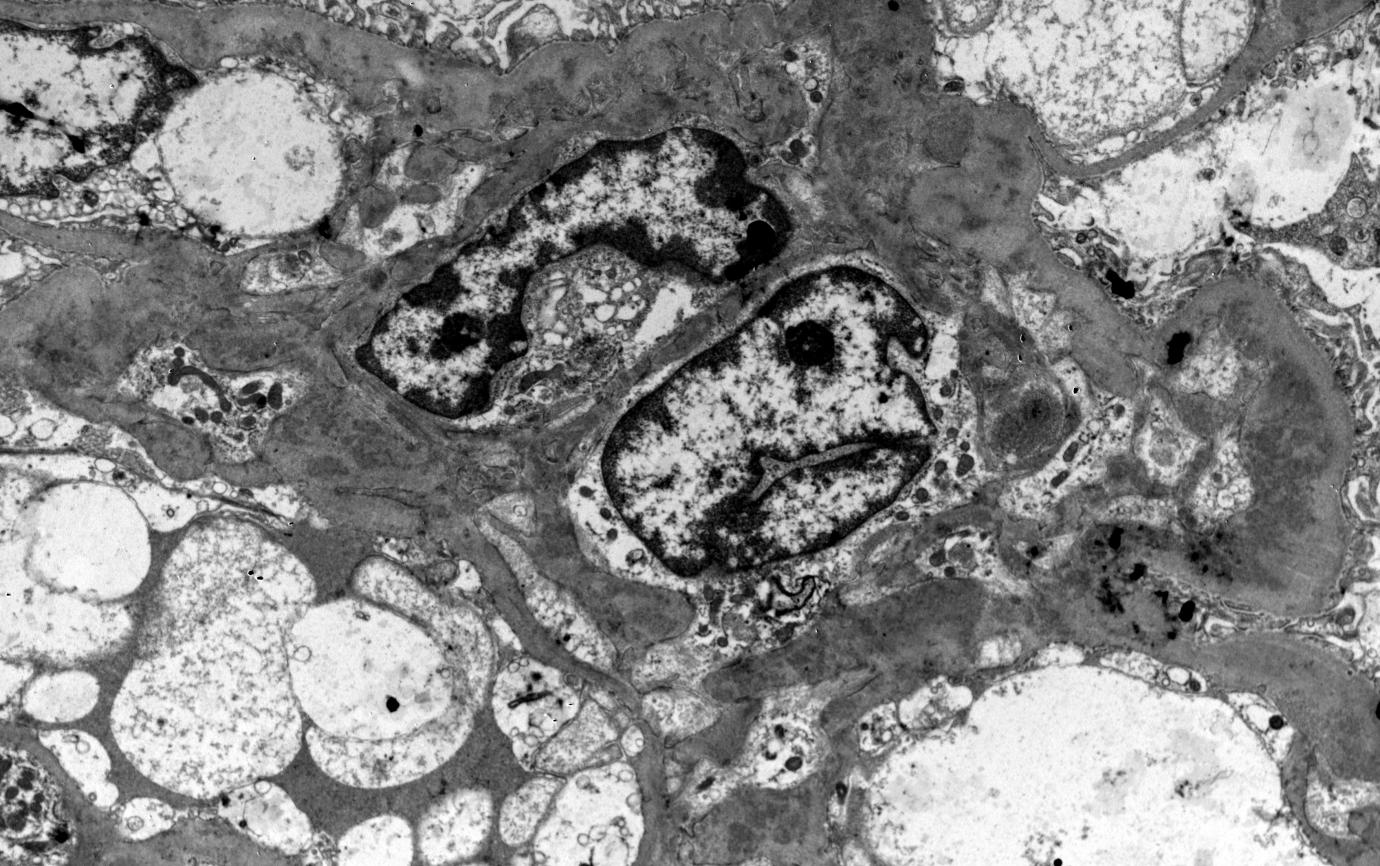
Numerous and large mesangial deposits, in a case of LN (Uranyl acetate X 4400).

Lupus nephritis is notorious for the large diversion of morphological patterns that disease may exhibit in histopathology. It is an immune-complex mediated nephritis, that is typically characterized by “full house” pattern in Immunofluorescence examination (that means Immunoglobulins IgG, IgA and IgM, complement components C3 and C1q, and kappa and lambda light chains, all are expressed in glomeruli **Figure 1**), while electron dense deposits are identified, sometimes in different glomerular locations, in ultrastructural examination by electron microscopy (EM) (**Figure 2**). Commonly, deposits may be found in different aspects of glomerulus in mesangial, subendothelial and/or subepithelial location/space and sometimes into tubular basement membranes or small vessel walls. Typical histological features also include the presence of tubuloreticular inclusions by EM examination, while in light microscopy examination, karyorrhexis, or large subendothelial deposits that form “wire loops” or “hyaline thrombi” are seen, especially in classes III and IV; the aforementioned histological features can also be of diagnostic importance. According to Kudose et al., full house pattern, intense C1q staining, extraglomerular deposits, combined subendothelial and subepithelial deposits, as well as endothelial tubuloreticular inclusions are important features of lupus nephritis, that can serve for the differential diagnosis from other glomerular diseases (the combination of these histological characteristics increases specificity and sensitivity of lupus nephritis diagnosis) (10).

Differential diagnosis of lupus nephritis includes Cryoglobulinemic glomerulonephritis, since both entities can display membranoproliferative pattern and “hyaline” thrombi into glomerular loops. In addition, lupus nephritis may show “organized” deposits by EM examination and sometimes can coexist with cryoglobulinemia. However, IgM immunoglobulin is usually dominant in Cryoglobulinemic glomerulonephritis (IgG or IgA immunoglobulins are expressed in a much lesser degree, or can be fainter), and usually there is no “full house” pattern. Furthermore, detection of cryoglobulin substructure in ultrastructural examination can be of importance in differential diagnosis. Differential diagnosis also includes rare cases of non-lupus nephritides with full house pattern, that mimic LN (11), comprising different disease entities, such as infection-related glomerulonephritis (e.g. endocarditis-related glomerulonephritis etc), membranous glomerulopathy associated with malignancy, “lupus-like” immune-complex mediated GN in HIV-infected patients (12) etc. In some of these cases that demonstrate full house pattern in Immunofluorescence, there is no specific evidence of any particular etiology and lupus serology may also be negative; however, an adequate and close follow-up of these patients is mandatory, since some of them may eventually show positive lupus serology in the future. Rarely, podocytopathies, such as minimal change disease, have also been noted in patients with SLE. ISN/RPS consensus classification states that “the renal biopsy findings, per se, cannot be used to establish a diagnosis of SLE”, thus pathology findings should always be combined with serology and clinical findings/symptoms, in order to establish a diagnosis of LN (13).

**Figure 3:**
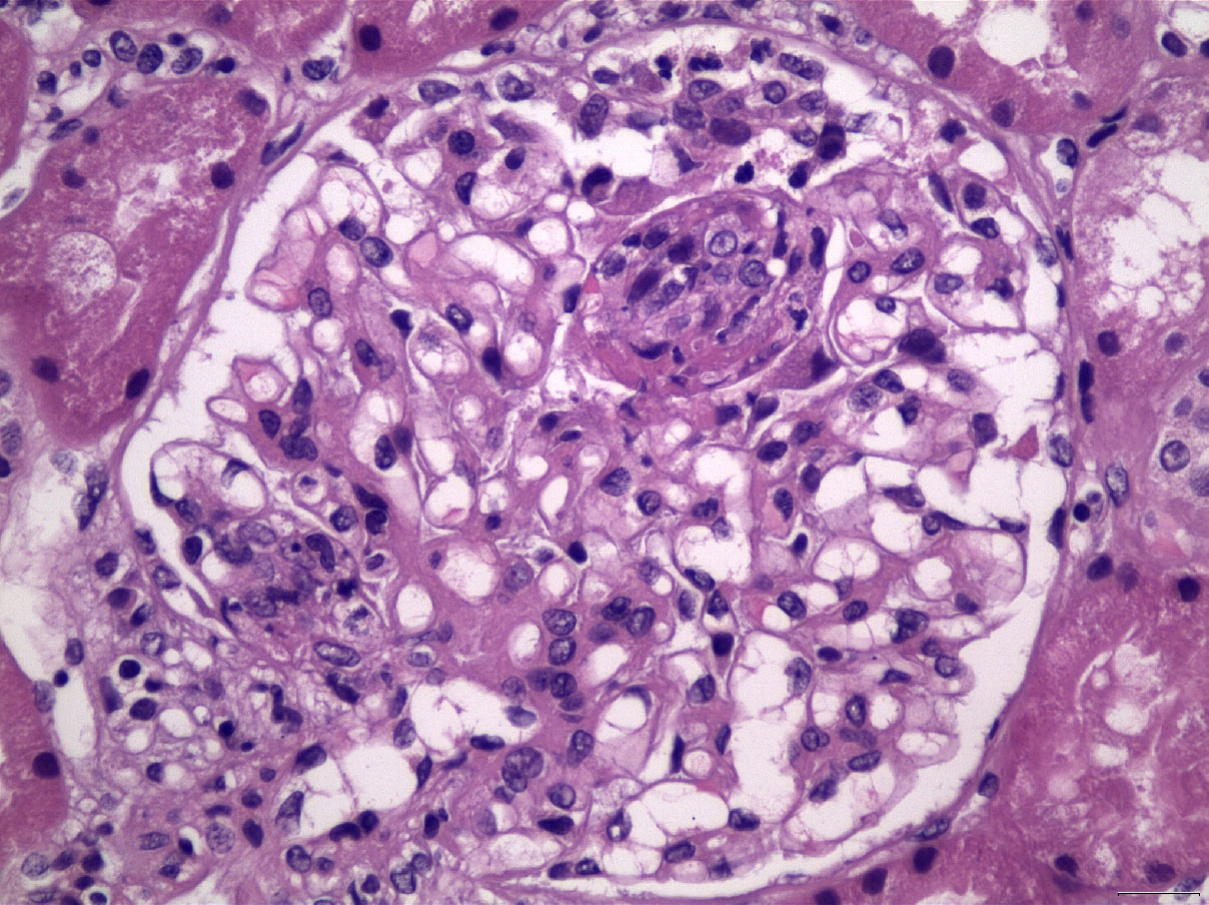
Segmental endocapillary proliferation, in association with a small focus of glomerular fibrinoid necrosis and karyorrhexis, in a case of LN Class III (H&E X400).

**Figure 4:**
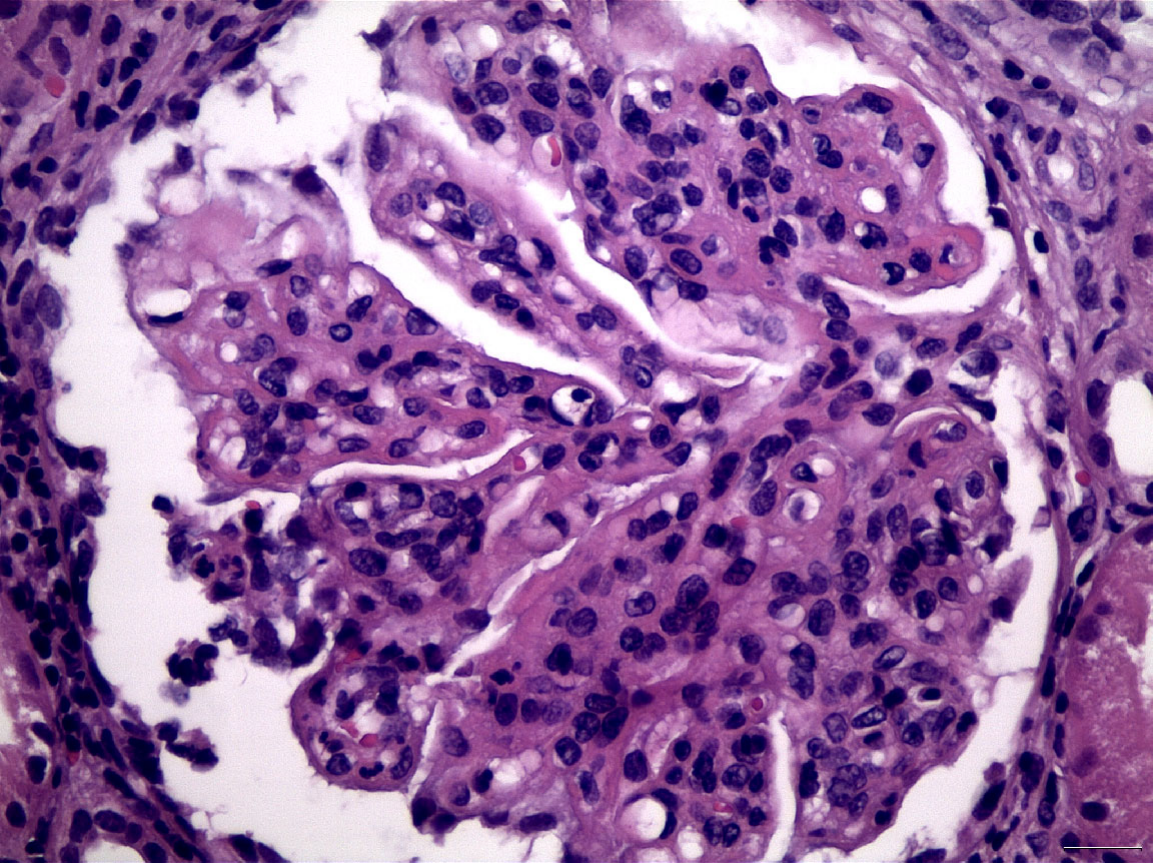
Membranoproliferative pattern, with lobulation of glomerular architecture, with mesangial expansion and proliferation, in association with capillary lumen occlusion by infiltrating inflammatory cells, including neutrophils, in a case of LN Class IV (H&E X400).

**Figure 5:**
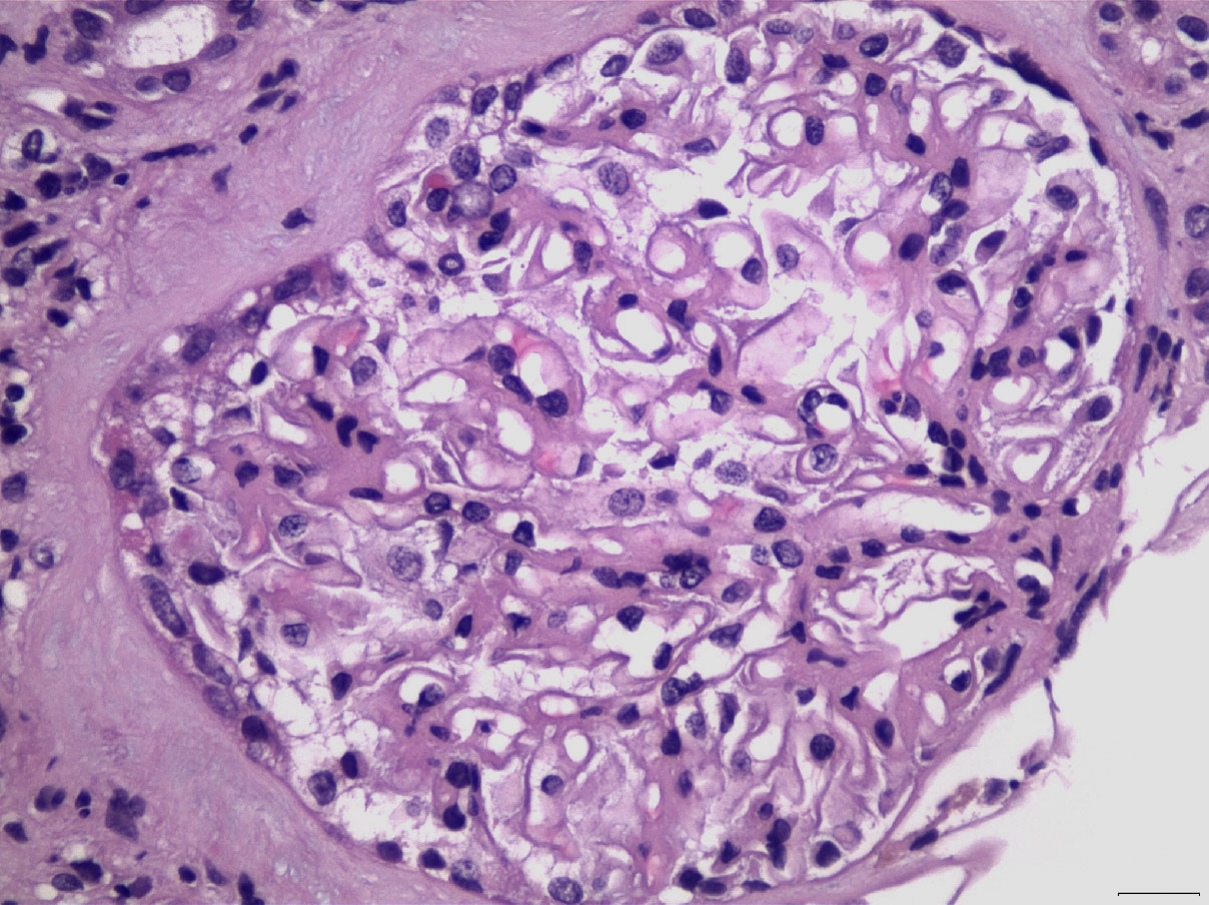
Glomerular capillary wall thickening, in association with mesangial expansion and proliferation, in a case of LN Class V (H&E X400).

Classification of LN divides histopathological patterns of glomerular injury into six “classes”, while the activity/chronicity indices should be applied in parallel in every biopsy, in order to determine the severity of the disease, the background chronicity, but also may provide useful guidance for the clinical therapeutic maneuvers, or even prognostic implications. The current ISN/RPS classification system for LN (13), has been modified in 2016, after a new consensus meeting and report (14). The “segmental” and “global” descriptive terms for class IV of LN, as well as the related indicators of activity and chronicity for class III and IV were eliminated in the latter modification. Furthermore, fibrinoid glomerular necrosis was proposed to be included in the activity index, as a separated and autonomous marker (in contrast with the previous Activity/Chronicity scheme), while some other minor proposals have also been suggested (14). According to the current classification, class I LN includes biopsies with normal histology on light microscopy, but “full house” pattern in Immunofluorescence examination, while class II includes biopsies with glomerular mesangial proliferation (without any endo- or extracapillary proliferation), and “full house” pattern in immunofluorescence microscopy. Class III LN (**Figure 3**) is characterized by focal endocapillary and/or extracapillary proliferation (<50% of glomeruli are involved, focal proliferative glomerulonephritis), while Class IV (**Figure 4**) includes biopsies with diffuse endocapillary and/or extracapillary proliferation (≥50% of glomeruli are involved, diffuse proliferative glomerulonephritis). Class V LN (**Figure 5**) comprises lupus membranous nephropathy with subepithelial deposits by LM and IF or EM, that may also show (or may not) mesangial proliferation. Class VI is characterized by advanced sclerosing LN (≥90% globally sclerosed glomeruli without activity in biopsy, while renal function is impaired, there is varying degree of proteinuria and urine sediment as well serology may be inactive at this phase, i.e., “burnt-out” lupus). The most common histopathological classes found in patients with SLE are III, IV and V, while mixed classes, combining Class III + V, or Class IV + V, can also be occasionally encountered (15).

A novel study by Bolognesi et al, suggested the presence of two main phenotypic forms/clusters of lupus nephritis, membranoproliferative-like and vasculitis-like, challenging the traditional classification, but further validation and confirmation from future studies are needed for establishing these observations (16).

Repeat of the renal biopsy during the disease course is essential for disease monitoring, since LN can display many faces, including class “transformation” (change from one class to another), a phenomenon which can be seen after therapy, but occasionally may be demonstrated in other situations. In addition, repeating renal biopsies can provide substantial information regarding chronicity, assessment of treatment response, or the detection of new flares of activity, in a chronic background. Furthermore, LN can be clinically “silent” and deceiving, and in some instances, histopathology may reveal the aggressive face of the disease, which may not be obvious in terms of clinical signs and symptoms. As expected, advanced chronicity has been linked with adverse kidney outcomes. The most important histological findings defining prognosis, that have also been recognized as risk factors for chronic kidney disease, are class type (Class IV), tubulointerstitial or vascular lesions or thrombotic microangiopathy and increased chronicity index (17).

## Definitions

There is no consensus agreement about the definitions of response, resistant disease and relapse in patients with LN, who are treated with immunosuppressive therapy and thus patients groups are heterogeneous in various studies (18). However, according to Kidney Disease Improving Global Outcomes (KDIGO) guidelines complete response translates into a decrease of proteinuria under 0.5 g/g (50 mg/mmol) assessed as the protein-creatinine ratio (PCR) from a 24-h urine collection and the enhancement or stabilization of renal function (+/- 10% of the baseline). Achievement of a partial response requires a decrease of proteinuria by at least 50% and under 3 g/g (300 mg/mmol) assessed as the PCR from a 24-h urine collection and the enhancement or stabilization of renal function (± 10%–15% of baseline). This definition refers to a time frame of 6-12 months after the initiation of immunosuppressive therapy, but complete response can take more than 12 months to be achieved (19). It is noteworthy that the term “response” is clinical and not the equivalent with the histopathologic term “remission”. A new kidney biopsy, which demonstrates the absence of active inflammation, is the only way a complete remission can be confirmed. However, in common clinical practice only selected patients undergo repeat kidney biopsy when a complete response of LN is achieved.

Not all patients with LN achieve a partial or complete clinical response after the initiation of immunosuppressive therapy. Resistant disease refers to the ineffectiveness of the initial immunosuppressive regimen to achieve a complete or a partial response, despite full adherence with the prescribed regimen. Yet, patients who have been given immunosuppressive therapy for LN may be shown resistant to it for other reasons, including nonadherence, inadequate scheme or dose, or duration of therapy and/or existence of genetic factors promoting chronic kidney disease. Generally, only a few patients remain unresponsive to both the two well-established therapy regimens of LN, namely a course of glucocorticoids plus cyclophosphamide or mycophenolate mofetil (MMF) (20).

Additionally, about 30% of patients with LN who initially achieved a complete response on immunosuppressive therapy and 60% of patients with partial response will subsequently experience a relapse (21). Relapse of LN is typically characterized by one or more of the following: active urine sediment, increased serum creatinine and/or increased urine protein excerption. Relapses can occur at any step of therapy, in patients on immunosuppressive therapy or during the decrease or cessation of immunosuppressants (22).

## Management of patients with lupus nephritis

Histopathological features of the kidney biopsy primarily guide the treatment of LN. All patients with an established diagnosis of SLE, including individuals with LN, should receive hydroxychloroquine (19). On the other hand, not all histopathological classes need immunosuppression.

### Class I & II

Patients with Class I (minimal mesangial) and Class II (mesangial proliferative) LN have a favorable renal prognosis and there is no justification for immunosuppressive therapy if extra-renal manifestations are absent. The only exception refers to patients with nephrotic syndrome attributed to lupus podocytopathy (5). In this occasion patients are treated with oral prednisolone 1mg/kg once daily (max. 80 mg) for 1 to 4 months which is gradually reduced after the achievement of remission (23). In any other case of Class I and Class II of LN, treatment is guided by extra-renal manifestations.

### Class III & IV

#### Induction

The treatment of Class III (focal) or Class IV (diffuse) LN consists of two phases: an initial phase in which anti-inflammatory and immunosuppressive agents are administered, followed by a second phase with long-term immunosuppressive therapy to ensure the sustained remission and avoid relapsing disease. The current perspectives for the management of LN are moving from sequential therapy i.e., strict separation of the two phases to an undivided approach, with combined therapies targeting multiple pathways of the immune system. In this regard, the duration of initial therapy may last as short as 3 months or as long as 1 year, but the average duration is approximately six months. The therapeutic goal of patients with the above histopathological classes is achievement of complete response. A delay in initiation of therapy must be avoided, as it may lead to irreversible kidney damage.

During the initial phase glucocorticoids are combined with either MMF or intravenous cyclophosphamide. A typical approach includes the administration of 0,5-1 mg/kg/d prednisolone (max. 80 mg/d) followed by a gradual tapering over a period of 3 to 6 months, which is widely adopted (24). In cases with more severe clinical and/or histopathological findings i.e. worsening kidney function or crescents presence in kidney biopsy, the administration of three intravenous daily pulses of 0,5-1g methylprednisolone is indicated (25). The adoption of intravenous cyclophosphamide has become the established norm in treating LN, improving kidney prognosis, and preventing the progression to ESKD. The typical National Institute of Health (NIH) regimen involves the administration of 0.5-1 g/m^2^ monthly doses of intravenous cyclophosphamide over a duration of 6 months (26). The alternative option is the Euro-Lupus regimen, which comprises the administration of 500 mg intravenous cyclophosphamide every 15 days over a span of 3 months, a remission-inducing regimen of low-dose intravenous cyclophosphamide (with a low cumulative cyclophosphamide dose of 3 grams) that leads to clinical outcomes comparable to those achieved with the full-dose regimen (27). Consequently, 6 months regimen is usually used only for the most severe forms of LN Intravenous cyclophosphamide is also indicated as the initial therapy in patients with low adherence to an oral regimen (19).

The other option includes the administration of glucocorticoids plus MMF, as no significantly different response rates between MMF versus cyclophosphamide was detected. Additionally, no significant differences were observed between the MMF and intravenous cyclophosphamide groups in terms of adverse events rates (28). MMF is administrated at a dose of 2-3 grams daily in two divided doses. For patients experiencing gastrointestinal side effects and cannot tolerate sufficient MMF doses, enteric-coated mycophenolate sodium (EC-MPS) is an alternative. While the guidelines for the optimal initial induction therapy remains ambiguous, MMF is typically favored for young patients with fertility concerns, given that cyclophosphamide could have adverse effects on fertility (19), if there is no indication of acute kidney injury and/or aggressive histopathological features in the renal biopsy. In this regard, according to EULAR recommendations, high-dose intravenous cyclophosphamide (0,5–0,75 g/m^2^ monthly for 6 months) is primarily administrated in patients with reduced glomerular filtration rate (GFR) and/or severe histopathological findings (23).

Rituximab, a monoclonal antibody targeting the CD20 antigen, and responsible for B cells depletion, is typically not used as initial induction therapy. This conclusion is based on a randomized trial participating 144 patients, where participants with class III or class IV LN receiving MMF plus corticosteroids, were randomly assigned to either rituximab (1,000 mg) or placebo. The trial concludes that no difference in rates of complete or partial response was detected between these groups (29).

Calcineurin inhibitors (CNI) alter immunity by affecting T cell function and also act as anti-proteinuric agents. Therefore, CNIs have been used in autoimmune kidney diseases with proteinuria such as LN. A series of trials, where the reduction of proteinuria was significantly higher in patients treated with regiment including CNI on top of standard therapy, established the use of tacrolimus as part of a triple regimen, widely known as “multitarget” (30). However, these data are limited and unable to establish the use of CNIs as initial therapy in all patients with severe LN. Only in patients who are unable to receive cyclophosphamide or MMF, or in case of pregnancy, tacrolimus may have a role in combination with glucocorticoids and azathioprine (31, 32). Besides, tacrolimus and CNIs in general are characterized by a narrow therapeutic range, need for frequent checking of drug levels, and their long-term use can lead to kidney injury and metabolic alternations. Voclosporin is a new entry, novel CNI, which does not require drug level monitoring and seems to lack many of the others CNIs adverse effects. Its efficacy and safety in active LN were evaluated in a phase III, randomized controlled multicenter trial participating 357 patients in which participants were randomized to receive voclosporin (23.7 mg twice daily) or placebo for a duration of 52 weeks, on top of MMF (2 g daily) and gradually tapered low-dose corticosteroids. At 52 weeks, higher rates of complete renal response (41% vs 23% odds ratio 2.65, 95% CI 1.64–4.27) were observed in Vocloporin group compared with placebo. Although the favorable side effect profile of voclosporin, hypertension and drug–drug interactions still occur (33). High cost of voclosporin is also a consideration, especially in limited resource settings (34). In United States voclosporin is Food and Drug Administration approved for the treatment of LN on top of MMF and glucocorticoids. However, in the new 2024 KDIGO guidelines for LN, initial therapy which includes a CNI (voclosporin, tacrolimus, or cyclosporine) on top of standard immunosuppressive therapy, may be indicated only in patients with an estimated glomerular filtration rate, (eGFR) > 45 ml/min/1.73 m^2^) and nephrotic range proteinuria and in patients, who have difficulties to tolerate standard-dose mycophenolic acid or are not suitable for cyclophosphamide-based regimens (19).

In the last years an immunosuppressive agent, belimumab, has arisen and it will potentially influence the initial phase of LN treatment. Belimumab is a IgG1-lambda monoclonal antibody which blocks the connection of soluble human B lymphocyte stimulator protein with receptors on B lymphocyte, resulting the apoptosis of B lymphocytes, which finally leads to the decrease of the autoimmune response. In a clinical trial participating patients with active LN, the addition of belimumab on top of induction therapy led to statistically significant better renal response compared to those patients who received standard therapy alone (35). In a *post hoc* subgroup analysis of this trial a greater proportion of patients achieved a primary efficacy renal response with belimumab versus placebo in the newly diagnosed [46.6% versus 37.2%, odds ratio 1.36 (95% CI 0.85–2.20)] and relapsed [36.0% versus 22.7%; odds ratio 2.31 (95% CI 1.07–5.01)] subgroups. Similarly, for complete renal remission [newly diagnosed: 33.8% versus 24.3%; odds ratio 1.49 (95% CI 0.88–2.51) and relapsed: [22.7% versus 10.7%; odds ratio 3.11 (95% CI 1.16–8.31)] (36). Another *post-hoc* analysis found that the higher rate of complete response with belimumab on top of standard therapy was limited to individuals with lower proteinuria (baseline urine PCR <3 g/g), while black race patients appear to have lower response rates (37). As a result, a triple immunosuppressive regimen including the standard of care plus belimumab is suggested for induction therapy in patients at high risk to progress to ESKD, although long term data to support this notion are lacking (19).

Recently, obinutuzumab, a new generation anti-CD20 monoclonal antibody, was tested as combinatory regiment for LN in the ongoing phase III REGENCY trial after the results of the phase II NOBILITY. In both trials, obinutuzumab was given at baseline and after six months of treatment. NOBILITY showed that in adult patients with active proliferative LN the addition of obinutuzumab on top of standard therapy with MPAAs and glucocorticoids resulted in higher complete renal response rates compared to placebo, while rates of serious adverse events did not differ between the Obinutuzumab and control group (38).

Furthermore, anifrolumab, a human monoclonal antibody against the type I interferon receptor, was approved for active extra-renal SLE after completion of trials, which however excluded active LN (39). Anifrolumab has been tested in a phase 2 clinical trial where 147 participants were randomized into three groups (anifrolumab 300 mg, anifrolumab 900 mg, or placebo) on top of MMF standard-of-care therapy. In this trial patients on anifrolumab appeared to have a higher renal response rate (45.5% vs. 31.1%) (40), which lead to the ongoing phase III trial testing anifrolumab as an add-on to standard immunosuppression on patients with biopsy-proven LN.

#### Management of resistant disease

The use of alternative therapy is suggested for patients with focal or diffuse LN, who show resistance to initial therapy. Patients who do not respond to cyclophosphamide are switched to MMF and vice versa. Although the lack of randomized trials, the use of rituximab to patients with resistant LN has showed favorable results in observational studies (41, 42). In a meta-analysis of 223 patients with refractory LN, response rates for partial and complete response was 27% and 51% respectively (43). Thus, KDIGO suggests the addition of rituximab or other biologic therapies i.e. belimumab, extended course of iv cyclophosphamide or enrollment in clinical trials when is feasible (19).

#### Maintenance therapy

After induction therapy, the next phase of LN treatment is aiming to prevent relapse. The length of maintenance therapy is 3 to 5 years and ideally it consists of 2gr MMF daily in two divided doses (23). According to the ALMS maintenance trial, MMF has shown better results than azathioprine in relapse prevention in patients with LN (44). However, azathioprine is still suggested for patients who seek pregnancy, or for patients who are incapable to tolerate MMF, in a dose of 2mg/kg per day (max dose 200mg/day) Oral prednisolone in a low dose of 0,05-0,2 mg/kg is continued in maintenance therapy in most cases and discontinuation should be considered only in patients who have maintained a complete renal response for more than 12 months (19). Patients who receive a triple immunosuppression regiment, which includes belimumab or CNI, can continue it in maintenance. However, in a study, where tacrolimus as subsequent therapy was tested, rates of relapse, serum creatinine and eGFR did not differ significantly between patients on tacrolimus and the control group (45).

#### Treatment of relapse

After a relapse, we suggest treating patients with the same immunosuppressive regimen that led to the remission at the initial phase of the disease. There are special concerns about cumulative cyclophosphamide dose in patients with frequent relapses or about infertility of young patients which may lead to the selection of the alternative of MMF (23, 24). The use of rituximab as induction therapy in relapses is lacking of randomized trials, although observational studies and case reports have shown favorable results (46, 47).

As far as belimumab is concerned, in a *post hoc* subgroup analysis of the BLISS-LN trial, a greater proportion of relapsing patients achieved a primary efficacy renal response with belimumab versus placebo and a complete renal remission. Moreover, kidney-related events or LN flares were significantly fewer in belimumab group versus placebo group (36). As a result, a triple immunosuppressive regimen including belimumab is clearly suggested for patients with repeated kidney flares (19).

### Class V (lupus membranous nephropathy)

Most of the patients with this histopathological class manifest nephrotic syndrome or nephrotic range proteinuria. The treatment of lupus patients with nephrotic syndrome due to lupus membranous nephropathy should involve immunosuppressive therapy. The deterioration of renal function or the preservation of nephrotic range proteinuria after the administration and titration of renin-angiotensin system blockers are also indications for immunosuppression. Decisions on treating with immunosuppressive medications patients with non-nephrotic proteinuria >1 g/24h need to be individualized, after considering the risk associated with the deterioration of the of kidney disease and the risks of treatment (19).

The general scheme of treatment consists of glucocorticoids in addition to MMF or cyclophosphamide or CNI or rituximab. Among the aforementioned treatments has been observed similar efficacy, although it has been shown that the treatment with MMF is likely to have a better safety profile, and the other options are preserved for patients who do not tolerate MMF or have contraindications. Calcineurin inhibitors, i.e., cyclosporine or tacrolimus, should be administrated with caution to patients with impaired renal function taking into consideration the potential for nephrotoxicity. KDIGO and the EULAR guidelines state that, MMF is the indicated first line treatment in these patients; the dose of MMF and cyclophosphamide is identical as for the treatment of LN class III and class IV. In cases where MMF is found to be not an effective treatment, intravenous cyclophosphamide may be administrated to induce long-term remission (28). As it happens to patients with primary membranous nephropathy, cyclophosphamide regimens are related with lower relapses rates (48).

Other alternative treatments include CNIs or rituximab, especially in patients who have already received high cumulative doses of cyclophosphamide or fulfill other contraindications (49). Cyclosporine, when administrated, has an initial dose scheme at 3-5 mg/kg/d in two divided doses and tacrolimus at 0,05-0,1mg/kg/d in two divided doses. Whole blood measurement is required for cyclosporine or tacrolimus levels and is necessary to be taken throughout this therapy. The expected range of results for cyclosporin is 100-200 ng/ml for C1 (cyclosporin levels before receiving the dose) and 600-800 ng/ml for C2 (cyclosporine levels 2 hours after receiving the dose), while for tacrolimus the expected range is 4-6 ng/ml, before receiving it (trough levels). Voclosporin has been also used in patients affected by lupus membranous nephropathy (LMN) in combination with MMF and glucocorticoids in AURORA 1 study. A subgroup analysis showed a trend of improved renal outcome in the small group of patients with pure LMN taking voclosporin, although not statistically significant (33). The main advantage with voclosporin, which is administrated in a dose of 23.7 mg twice daily, is that monitoring of blood levels is not required.

Although belimumab is an approved treatment for LN, due to limited experience and lack of long-term data is not yet a well-established approach when treating LMN. The efficacy of belimumab when combined with standard therapy in patients with LMN has been tested in BLISS-LN trial, which included a limited number of patients with LMN (72 patients with pure LMN and 116 with concurrent focal or diffuse LN plus LMN) (35). Patients with histopathological findings of concurrent LMN and Class III or Class IV of LN are treated with a similar approach as implemented for the patients with Class III or Class IV LN alone (24).

### Class VI (advanced sclerosing lupus nephritis)

Class VI LN is related to global sclerosis of a percentage greater than 90% of glomeruli. It has been observed that immunosuppressive therapy might not be effective to treat these patients and it is likely to result in adverse effects. Because of that observation, these patients should be treated as CKD, with monitoring and control of blood pressure levels and other comorbidities to decrease proteinuria and prepare for kidney replacement therapy when needed.

## General measures

As part of the holistic treatment of patients with LN, general supportive measures are significant as are when treating patients with glomerulonephritis. These measures include dietary restrictions as for example salt intake to <5 g/day and protein intake <0,8g/kg/day for patients with CKD levels eGFR<60 ml/min/1,73m^2^. This dietary recommendation is advised to be combined with a lifestyle approach that includes among others normal physical activity, optimal body weight and smoking cessation. The pharmaceutical approach involves using angiotensin-converting-enzyme inhibitors or alternatively angiotensin receptor blockers which should be administrated to the maximally tolerated daily dose. These interventions aim to minimize proteinuria and concomitantly control blood pressure levels (<120-130/80mmHg). It also involves treating hyperlipidemia using statins when required and providing thrombosis prophylaxis for patients with hypoalbuminemia as well as trimethoprim/sulfamethoxazole or Atovaquone as prophylaxis for pneumocystis jirovecii pneumonia. Finally, special care should be paid to minimize bone loss and prevent osteoporosis as a result of the glucocorticoid treatment and to the avoidance of superimposing kidney injury/drug nephrotoxicity (50). Data from a clinical trial testing finerenone in non-diabetic CKD patients, on top of standard treatment are also pending to prove if finerenone plus renin angiotensin system inhibitors slow the progression of CKD and decrease cardiovascular events, as it is already established for patients with diabetic kidney disease (51).

### SGLT2 inhibitors

Except for renin angiotensin system inhibitors, the pharmaceutical intervention with sodium-glucose co-transporter-2 (SGLT2) inhibitors has shown SLE-nonspecific effects by altering LN progression affecting non-immune mechanisms. Yet, SGLT2 inhibitors have already established benefits in slowing the progression of CKD and contribute in cardiovascular protection in addition to the standard renin angiotensin system blockade in non-diabetic CKD patients (52, 53). *Post hoc* studies have shown this effect in both IgA nephropathy and podocytopathies (54, 55). Patients with LN and antineutrophil cytoplasmic autoantibodies-associated vasculitis were included in the EMPA-Kidney trial, which compared the SGLT2 inhibitor empagliflozin vs placebo, but the results from this dedicated subgroup of patients are not yet published. The findings of a randomized controlled trial suggest 7.4 more years of survival free of kidney failure with the combination of renin angiotensin system inhibitors and SGLT2 inhibitors in patients with albuminuric CKD without diabetes (56). With these pharmaceutical interventions an additional and beneficial diuretic impact on nephrotic syndrome might be expected. Thus, when they are used on top of standard diuretic therapy, careful monitoring for hypovolemia is needed.

## Patients with ESKD and kidney transplantation

As referred above, a percentage of 10-30% of patients diagnosed with LN progress to ESKD. The treatment of patients who end up in ESKD can be approached with either kidney transplantation or hemodialysis/peritoneal dialysis.

Among the treatments mentioned above, kidney transplantation remains by far the ideal modality, as it has been shown that kidney transplantation has the optimum prognosis and thus it is preferred when compared to hemodialysis or peritoneal dialysis (57). New evidence suggests that in case there are no extrarenal manifestations contraindicated surgery, a preemptive transplantation is also recommended to be performed (58, 59).

Analyzing the United Network for Organ Sharing files has shown that the recurrence rate of LN at the kidney graft concerned the 2,4% of cases (60). The histopathologic image is milder possibly due to ongoing immunosuppression and most patients do not even require changing their antirejection immunosuppression regiment. There no evidence that the presence of serologic disease activity at the time of transplantation is correlated with transplant outcome or disease recurrence (61). On the other hand, all patients with SLE should be controlled for the presence of antiphospholipid antibodies before transplantation. This is because the presence of antiphospholipid antibodies has been associated with increased risk for thrombotic events, including the thrombotic microangiopathy in the allograft (62).

The survival rates and mortality among SLE patients on hemodialysis or on peritoneal dialysis are not shown to have any difference (63).. Chronic hemodialysis in patients with SLE has been associated with decreased clinical and serologic lupus activity (64).

## Discussion

As seen above, although the medical community has made a lot of efforts to improve the prognosis of LN, approximately 10-30% of patients, the majority of them being relatively young, will eventually progress to ESKD, a percentage which is inappropriately high. Hence, the need to improve the outcomes has not been met. The introduction of the above-mentioned new agents has unquestionably opened new roads and new therapeutic possibilities. The added effect of the combined regimens has increased renal remission rates at a proportion of 10-20%, as seen from the recent belimumab and voclosporin studies (33, 35). However, clinicians managing patients with LN always consider the fragility of this population, most of them being women at reproductive age, who can certainly benefit from years free of ESKD. Another point is that a lot of patients will achieve the renal goals of remission without the use of combination therapies and thus selection criteria are required in order to avoid overtreatment. The problem is that there are no specific biomarkers to predict outcomes and/or express the underlying immunologic activity. One approach could be the one reported by Mejia-Vilet et al. who proposed patient stratification by waiting the first 3 months of standard therapy and by accessing then the level of proteinuria. If proteinuria is not reduced beyond 25%, they propose the addition of belimumab or voclosporin (65). In regard to refractory or relapsing LN the use of combination therapy is almost mandatory. The high cost of the new medications should be considered in the context of the fact that the cost of lifelong dialysis is definitely higher, while the quality of life is dramatically decreasing in the setting of ESKD. As always, national authorities and regulatory mechanisms must prioritize the medical needs, but in this case the comparison with the financial burdens of dialysis obviously favors the former.

## Author contributions

EX: Writing – original draft. KD: Writing – original draft. GL: Writing – original draft. SL: Conceptualization, Writing – review & editing.

## References

[1] Goulielmos GN Zervou MI Vazgiourakis VM Ghodke-Puranik Y Garyfallos A Niewold TB. The genetics and molecular pathogenesis of systemic lupus erythematosus (SLE) in populations of different ancestry. Gene. (2018) 668:59–72. doi: 10.1016/j.gene.2018.05.041 29775752

[2] Almaani S Meara A Rovin BH. Update on lupus nephritis. Clin J Am Soc Nephrol. (2017) 12:825–35. doi: 10.2215/CJN.05780616 PMC5477208 27821390

[3] Hanly JG O’Keeffe AG Su L Urowitz MB Romero-Diaz J Gordon C. The frequency and outcome of lupus nephritis: results from an international inception cohort study. Rheumatol (Oxford). (2016) 55:252–62. doi: 10.1093/rheumatology/kev311 PMC4939728 26342222

[4] Yu F Wu LH Tan Y Li LH Wang CL Wang WK. Tubulointerstitial lesions of patients with lupus nephritis classified by the 2003 International Society of Nephrology and Renal Pathology Society system. Kidney Int. (2010) 77:820–9. doi: 10.1038/ki.2010.13 20182417

[5] Kraft SW Schwartz MM Korbet SM Lewis EJ. Glomerular podocytopathy in patients with systemic lupus erythematosus. J Am Soc Nephrol. (2005) 16:175–9. doi: 10.1681/ASN.2004050350 15548564

[6] Kwok SK Ju JH Cho CS Kim HY Park SH. Thrombotic thrombocytopenic purpura in systemic lupus erythematosus: risk factors and clinical outcome: a single centre study. Lupus. (2009) 18:16–21. doi: 10.1177/0961203308094360 19074164

[7] Abdellatif AA Waris S Lakhani A Kadikoy H Haque W Truong LD. True vasculitis in lupus nephritis. Clin Nephrol. (2010) 74:106–12. doi: 10.5414/CNP74106 20630130

[8] Anders HJ Saxena R Zhao MH Parodis I Salmon JE Mohan C. Lupus nephritis. Nat Rev Dis Primers. (2020) 6:7. doi: 10.1038/s41572-019-0141-9 31974366

[9] Rovin BH Caster DJ Cattran DC Gibson KL Hogan JJ Moeller MJ. Management and treatment of glomerular diseases (part 2): conclusions from a Kidney Disease: Improving Global Outcomes (KDIGO) Controversies Conference. Kidney Int. (2019) 95:281–95. doi: 10.1016/j.kint.2018.11.008 30665569

[10] Kudose S Santoriello D Bomback AS Stokes MB D’Agati VD Markowitz GS. Sensitivity and specificity of pathologic findings to diagnose lupus nephritis. Clin J Am Soc Nephrol. (2019) 14:1605–15. doi: 10.2215/CJN.01570219 PMC6832038 31653670

[11] Rijnink EC Teng YKO Kraaij T Wolterbeek R Bruijn JA Bajema IM. Idiopathic non-lupus full-house nephropathy is associated with poor renal outcome. Nephrol Dial Transplant. (2017) 32:654–62. doi: 10.1093/ndt/gfx020 28340077

[12] Kofotolios I Tsiakas S Skalioti C Kapsia E Liapis G Marinaki S. Treatment of HIV-associated lupus-like membranous nephropathy with tacrolimus: A case report and review of the literature. Life (Basel). (2023) 13:641. doi: 10.3390/life13030641 36983799 PMC10053887

[13] Weening JJ D’Agati VD Schwartz MM Seshan SV Alpers CE Appel GB. The classification of glomerulonephritis in systemic lupus erythematosus revisited. J Am Soc Nephrol. (2004) 15:241–50. doi: 10.1097/01.ASN.0000108969.21691.5D 14747370

[14] Bajema IM Wilhelmus S Alpers CE Bruijn JA Colvin RB Cook HT. Revision of the International Society of Nephrology/Renal Pathology Society classification for lupus nephritis: clarification of definitions, and modified National Institutes of Health activity and chronicity indices. Kidney Int. (2018) 93:789–96. doi: 10.1016/j.kint.2017.11.023 29459092

[15] Jennette JC Olson JL Fred GS D’Agati VD. Renal disease in systemic lupus erythematous, mixed connective tissue disease, Sjogren syndrome and rheumatoid arthritis. In: D’Agati VD Stokes BM, editors. Heptinstall’s pathology of the kidney, 7th ed, vol. 1 . Lippincott Williams & Wilkins, Philadelphia, PA, USA (2014).

[16] Bolognesi MM Capitoli G Galimberti S Cattoretti G Bajema I Bruijn JA. Dissecting the histological features of lupus nephritis highlights new common patterns of injury in class III/IV. Ann Rheum Dis. (2022) 81:1704–11. doi: 10.1136/ard-2022-222620 35940846

[17] Rodríguez-Almaraz E Gutiérrez-Solís E Rabadán E Rodríguez P Carmona L Morales E. Something new about prognostic factors for lupus nephritis? A systematic review. Lupus. (2021) 30:2256–67. doi: 10.1177/09612033211061475 34907831

[18] Weidenbusch M Bai Y Eder J Anders HJ. Lupus nephritis trials network. Refractory lupus nephritis: survey Lupus. (2019) 28:455–64. doi: 10.1177/0961203319828516 30755142

[19] Rovin BH Ayoub IM Chan TM Liu ZH Mejía-Vilet JM Floege J. KDIGO 2024 clinical practice guideline for the management of LUPUS NEPHRITIS. Kidney Int. (2024) 105:S1–69. doi: 10.1016/j.kint.2023.09.002 38182286

[20] Anders HJ Hiepe F. Treatment options for refractory lupus nephritis. Clin J Am Soc Nephrol. (2019) 14:653–5. doi: 10.2215/CJN.03230319 PMC6500949 30979714

[21] Illei GG Austin HA Crane M Collins L Gourley MF Yarboro CH. Combination Therapy with Pulse Cyclophosphamide plus Pulse Methylprednisolone Improves Long-Term Renal Outcome without Adding Toxicity in Patients with Lupus Nephritis. Ann Intern Med. (2001) 135:248–57. doi: 10.7326/0003-4819-135-4-200108210-00009 11511139

[22] Illei GG Takada K Parkin D Austin HA Crane M Yarboro CH. Renal flares are common in patients with severe proliferative lupus nephritis treated with pulse immunosuppressive therapy: long-term followup of a cohort of 145 patients participating in randomized controlled studies. Arthritis Rheumatol. (2002) 46:995–1002. doi: 10.1002/art.10142 11953977

[23] Fanouriakis A Kostopoulou M Cheema K Anders HJ Aringer M Bajema I. 2019 Update of the Joint European League Against Rheumatism and European Renal Association-European Dialysis and Transplant Association (EULAR/ERA-EDTA) recommendations for the management of lupus nephritis. Ann Rheum Dis. (2020) 79:713–23. doi: 10.1136/annrheumdis-2020-216924 32220834

[24] Rovin BH Adler SG Barratt J Bridoux F Burdge KA Chan TM. KDIGO 2021 clinical practice guideline for the management of glomerular diseases. Kidney Int. (2021) 100:S1–276. doi: 10.1016/j.kint.2021.05.021 34556256

[25] Boumpas DT Austin HA Vaughn EM Klippel JH Steinberg AD Yarboro CH. Controlled trial of pulse methylprednisolone versus two regimens of pulse cyclophosphamide in severe lupus nephritis. Lancet. (1992) 340:741–5. doi: 10.1016/0140-6736(92)92292-N 1356175

[26] Austin HA Klippel JH Balow JE le Riche NG Steinberg AD Plotz PH. Therapy of lupus nephritis. Controlled trial of prednisone and cytotoxic drugs. N Engl J Med. (1986) 314:614–9. doi: 10.1056/NEJM198603063141004 3511372

[27] Houssiau FA Vasconcelos C D’Cruz D Sebastiani GD Garrido Ed E de R Danieli MG. Immunosuppressive therapy in lupus nephritis: the Euro-Lupus Nephritis Trial, a randomized trial of low-dose versus high-dose intravenous cyclophosphamide. Arthritis Rheumatol. (2002) 46:2121–31. doi: 10.1002/art.10461 12209517

[28] Appel GB Contreras G Dooley MA Ginzler EM Isenberg D Jayne D. Mycophenolate mofetil versus cyclophosphamide for induction treatment of lupus nephritis. J Am Soc Nephrol. (2009) 20:1103–12. doi: 10.1681/ASN.2008101028 PMC2678035 19369404

[29] Rovin BH Furie R Latinis K Looney RJ Fervenza FC Sanchez-Guerrero J. Efficacy and safety of rituximab in patients with active proliferative lupus nephritis: the Lupus Nephritis Assessment with Rituximab study. Arthritis Rheumatol. (2012) 64:1215–26. doi: 10.1002/art.34359 22231479

[30] Liu Z Zhang H Liu Z Xing C Fu P Ni Z. Multitarget therapy for induction treatment of lupus nephritis: a randomized trial. Ann Intern Med. (2015) 162:18–26. doi: 10.7326/M14-1030 25383558

[31] Ichinose K Sato S Kitajima Y Horai Y Fujikawa K Umeda M. The efficacy of adjunct tacrolimus treatment in pregnancy outcomes in patients with systemic lupus erythematosus. Lupus. (2018) 27:1312–20. doi: 10.1177/0961203318770536 29665758

[32] Webster P Wardle A Bramham K Webster L Nelson-Piercy C Lightstone L. Tacrolimus is an effective treatment for lupus nephritis in pregnancy. Lupus. (2014) 23:1192–6. doi: 10.1177/0961203314540353 24928830

[33] Rovin BH Teng YKO Ginzler EM Arriens C Caster DJ Romero-Diaz J. Efficacy and safety of voclosporin versus placebo for lupus nephritis (AURORA 1): a double-blind, randomised, multicentre, placebo-controlled, phase 3 trial. Lancet. (2021) 397:2070–80. doi: 10.1016/S0140-6736(21)00578-X 33971155

[34] Kale A Shelke V Lei Y Gaikwad AB Anders HJ. Voclosporin: unique chemistry, pharmacology and toxicity profile, and possible options for implementation into the management of lupus nephritis. Cells. (2023) 12:2440. doi: 10.3390/cells12202440 37887284 PMC10605893

[35] Furie R Rovin BH Houssiau F Malvar A Teng YKO Contreras G. Two-year, randomized, controlled trial of belimumab in lupus nephritis. N Engl J Med. (2020) 383:1117–28. doi: 10.1056/NEJMoa2001180 32937045

[36] Anders HJ Furie R Malvar A Zhao MH Hiromura K Weinmann-Menke J. Effect of belimumab on kidney-related outcomes in patients with lupus nephritis: *post hoc* subgroup analyses of the phase 3 BLISS-LN trial. Nephrol Dial Transplant. (2023) 38:2733–42. doi: 10.1093/ndt/gfad167 PMC10689192 37463054

[37] Rovin BH Furie R Teng YKO Contreras G Malvar A Yu X. A secondary analysis of the Belimumab International Study in Lupus Nephritis trial examined effects of belimumab on kidney outcomes and preservation of kidney function in patients with lupus nephritis. Kidney Int. (2022) 101:403–13. doi: 10.1016/j.kint.2021.08.027 34560137

[38] Furie RA Aroca G Cascino MD Garg JP Rovin BH Alvarez A. B-cell depletion with obinutuzumab for the treatment of proliferative lupus nephritis: a randomised, double-blind, placebo-controlled trial. Ann Rheum Dis. (2022) 81:100–7. doi: 10.1136/annrheumdis-2021-220920 PMC8762029 34615636

[39] Morand EF Furie R Tanaka Y Bruce IN Askanase AD Richez C. Trial of anifrolumab in active systemic lupus erythematosus. New Engl J Med. (2020) 382:211–21. doi: 10.1056/NEJMoa1912196 31851795

[40] Jayne D Rovin B Mysler EF Furie RA Houssiau FA Trasieva T. Phase II randomised trial of type I interferon inhibitor anifrolumab in patients with active lupus nephritis. Ann Rheum Dis. (2022) 81:496–506. doi: 10.1136/annrheumdis-2021-221478 35144924 PMC8921596

[41] Weidenbusch M Römmele C Schröttle A Anders HJ. Beyond the LUNAR trial. Efficacy of rituximab in refractory lupus nephritis. Nephrol Dial Transplant. (2013) 28:106–11. doi: 10.1093/ndt/gfs285 22764193

[42] Vigna-Perez M Hernández-Castro B Paredes-Saharopulos O Portales-Pérez D Baranda L Abud-Mendoza C. Clinical and immunological effects of Rituximab in patients with lupus nephritis refractory to conventional therapy: a pilot study. Arthritis Res Ther. (2006) 8:R83. doi: 10.1186/ar1954 16677395 PMC1526618

[43] Alshaiki F Obaid E Almuallim A Taha R El-haddad H Almoallim H. Outcomes of rituximab therapy in refractory lupus: A meta-analysis. Eur J Rheumatol. (2018) 5:118. doi: 10.5152/eurjrheumatol. 30185361 PMC6072690

[44] Dooley MA Jayne D Ginzler EM Isenberg D Olsen NJ Wofsy D. Mycophenolate versus azathioprine as maintenance therapy for lupus nephritis. New Engl J Med. (2011) 365:1886–95. doi: 10.1056/NEJMoa1014460 22087680

[45] Zhang H Liu Z Zhou M Liu Z Chen J Xing C. Multitarget therapy for maintenance treatment of lupus nephritis. J Am Soc Nephrol. (2017) 28:3671–8. doi: 10.1681/ASN.2017030263 PMC5698076 28760751

[46] Boletis JN Marinaki S Skalioti C Lionaki SS Iniotaki A Sfikakis PP. Rituximab and mycophenolate mofetil for relapsing proliferative lupus nephritis: a long-term prospective study. Nephrol Dial Transplant. (2009) 24:2157–60. doi: 10.1093/ndt/gfp002 19179411

[47] Melander C Sallée M Trolliet P Candon S Belenfant X Daugas E. Rituximab in severe lupus nephritis: early B-cell depletion affects long-term renal outcome. Clin J Am Soc Nephrol. (2009) 4:579–87. doi: 10.2215/CJN.04030808 PMC2653670 19261822

[48] Austin HA Illei GG Braun MJ Balow JE. Randomized, controlled trial of prednisone, cyclophosphamide, and cyclosporine in lupus membranous nephropathy. J Am Soc Nephrol. (2009) 20:901–11. doi: 10.1681/ASN.2008060665 PMC2663831 19297556

[49] Chavarot N Verhelst D Pardon A Caudwell V Mercadal L Sacchi A. Rituximab alone as induction therapy for membranous lupus nephritis: A multicenter retrospective study. Medicine. (2017) 96:e7429. doi: 10.1097/MD.0000000000007429 28682905 PMC5502178

[50] Anders HJ Fernandez-Juarez GM Vaglio A Romagnani P Floege J. CKD therapy to improve outcomes of immune-mediated glomerular diseases. Nephrol Dialysis Transplant. (2023) 38:ii50–7. doi: 10.1093/ndt/gfad069 37218706

[51] Bakris GL Agarwal R Anker SD Pitt B Ruilope LM Rossing P. Effect of finerenone on chronic kidney disease outcomes in type 2 diabetes. N Engl J Med. (2020) 383:2219–29. doi: 10.1056/NEJMoa2025845 33264825

[52] The EMPA-KIDNEY Collaborative Group Herrington WG Staplin N Wanner C Green JB Hauske SJ. Empagliflozin in patients with chronic kidney disease. N Engl J Med. (2023) 388:117–27. doi: 10.1056/NEJMoa2204233 PMC7614055 36331190

[53] Heerspink HJL Stefánsson BV Correa-Rotter R Chertow GM Greene T Hou FF. Dapagliflozin in patients with chronic kidney disease. N Engl J Med. (2020) 383:1436–46. doi: 10.1056/NEJMoa2024816 32970396

[54] Wheeler DC Jongs N Stefansson BV Chertow GM Greene T Hou FF. Safety and efficacy of dapagliflozin in patients with focal segmental glomerulosclerosis: a prespecified analysis of the dapagliflozin and prevention of adverse outcomes in chronic kidney disease (DAPA-CKD) trial. Nephrol Dial Transplant. (2022) 37:1647–56. doi: 10.1093/ndt/gfab335 PMC9395378 34850160

[55] Wheeler DC Toto RD Stefánsson BV Jongs N Chertow GM Greene T. A pre-specified analysis of the DAPA-CKD trial demonstrates the effects of dapagliflozin on major adverse kidney events in patients with IgA nephropathy. Kidney Int. (2021) 100:215–24. doi: 10.1016/j.kint.2021.03.033 33878338

[56] Vart P Vaduganathan M Jongs N Remuzzi G Wheeler DC Hou FF. Estimated lifetime benefit of combined RAAS and SGLT2 inhibitor therapy in patients with albuminuric CKD without diabetes. Clin J Am Soc Nephrol. (2022) 17:1754–62. doi: 10.2215/CJN.08900722 PMC9718016 36414316

[57] Jorge A Wallace ZS Lu N Zhang Y Choi HK. Renal transplantation and survival among patients with lupus nephritis. Ann Intern Med. (2019) 170:240–7. doi: 10.7326/M18-1570 PMC6739121 30665236

[58] Parikh SV Almaani S Brodsky S Rovin BH. Update on lupus nephritis: core curriculum 2020. Am J Kidney Dis. (2020) 76:265–81. doi: 10.1053/j.ajkd.2019.10.017 32220510

[59] Plantinga LC Patzer RE Drenkard C Kramer MR Klein M Lim SS. Association of time to kidney transplantation with graft failure among U.S. Patients with end-stage renal disease due to lupus nephritis. Arthritis Care Res (Hoboken). (2015) 67:571–81. doi: 10.1002/acr.22482 PMC4370810 25251922

[60] Contreras G Mattiazzi A Guerra G Ortega LM Tozman EC Li H. Recurrence of lupus nephritis after kidney transplantation. J Am Soc Nephrol. (2010) 21:1200–7. doi: 10.1681/ASN.2009101093 PMC3152228 20488956

[61] Yap KS Urowitz MB Mahood Q Medina-Rosas J Sabapathy A Lawson D. The utility of lupus serology in predicting outcomes of renal transplantation in lupus patients: Systematic literature review and analysis of the Toronto lupus cohort. Semin Arthritis Rheumatol. (2017) 46:791–7. doi: 10.1016/j.semarthrit.2016.09.008 27769590

[62] Ames PR Merashli M Bucci T Gentile F Delgado-Alves J. Antiphospholipid antibodies and renal transplant: A systematic review and meta-analysis. Semin Arthritis Rheumatol. (2019) 48:1041–52. doi: 10.1016/j.semarthrit.2018.10.016 30449651

[63] Contreras G Pagan J Chokshi R Virmani S Diego JM Byers P. Comparison of mortality of ESRD patients with lupus by initial dialysis modality. Clin J Am Soc Nephrol. (2014) 9:1949–56. doi: 10.2215/CJN.02500314 PMC4220755 25189924

[64] Mojcik CF Klippel JH. End-stage renal disease and systemic lupus erythematosus. Am J Med. (1996) 101:100–7. doi: 10.1016/S0002-9343(96)00074-5 8686702

[65] Mejia-Vilet JM Malvar A Arazi A Rovin BH. The lupus nephritis management renaissance. Kidney Int. (2022) 101:242–55. doi: 10.1016/j.kint.2021.09.012 34619230

